# Alteration of Ethanol Reward by Prior Mephedrone Exposure: The Role of Age and Matrix Metalloproteinase-9 (MMP-9)

**DOI:** 10.3390/ijms23042122

**Published:** 2022-02-14

**Authors:** Pawel Grochecki, Irena Smaga, Marta Marszalek-Grabska, Malgorzata Lopatynska-Mazurek, Tymoteusz Slowik, Ewa Gibula-Tarlowska, Ewa Kedzierska, Joanna Listos, Malgorzata Filip, Jolanta H. Kotlinska

**Affiliations:** 1 Department of Pharmacology and Pharmacodynamics, Medical University, Chodzki 4a, 20-093 Lublin, Poland; pawelgrochecki@umlub.pl (P.G.); gosia.lopatynska@gmail.com (M.L.-M.); ewa.gibula@umlub.pl (E.G.-T.); ewa.kedzierska@umlub.pl (E.K.); joanna.listos@umlub.pl (J.L.); 2 Department of Drug Addiction Pharmacology, Maj Institute of Pharmacology, Polish Academy of Sciences, Smetna 12, 31-343 Kraków, Poland; smaga@if-pan.krakow.pl (I.S.); mal.fil@if-pan.krakow.pl (M.F.); 3 Department of Experimental and Clinical Pharmacology, Medical University, Jaczewskiego 8b, 20-090 Lublin, Poland; marta.marszalek-grabska@umlub.pl; 4 Experimental Medicine Center, Medical University of Lublin, Jaczewskiego 8, 20-090 Lublin, Poland; tymoteusz.slowik@tlen.pl

**Keywords:** mephedrone, ethanol, conditioned place preference, minocycline, MMP-9, age

## Abstract

Mephedrone, a synthetic cathinone, is widely abused by adolescents and young adults. The aim of this study was to determine: (i) whether prior mephedrone exposure would alter ethanol reward and (ii) whether age and matrix metalloproteinase-9 (MMP-9) are important in this regard. In our research, male Wistar rats at postnatal day 30 (PND30) received mephedrone at the dose of 10 mg/kg, i.p., 3 times a day for 7 days. To clarify the role of MMP-9 in the mephedrone effects, one mephedrone-treated group received minocycline, as an MMP-9 antagonist. Animals were then assigned to conditioned place preference (CPP) procedure at PND38 (adolescent) or at PND69 (adult). After the CPP test (PND48/79), expression of dopamine D1 receptors (D1R), Cav1.2 (a subtype of L-type calcium channels), and MMP-9 was quantified in the rat ventral striatum (vSTR). The influence of mephedrone administration on the N-methyl-D-aspartate glutamate receptors (NMDAR) subunits (GluN1, GluN2A, and GluN2B) was then assessed in the vSTR of adult rats (only). These results indicate that, in contrast with adolescent rats, adult rats with prior mephedrone administration appear to be more sensitive to the ethanol effect in the CPP test under the drug-free state. The mephedrone effect in adult rats was associated with upregulation of D1R, NMDAR/GluN2B, MMP-9, and Cav1.2 signaling. MMP-9 appears to contribute to these changes in proteins expression because minocycline pretreatment blocked mephedrone-evoked sensitivity to ethanol reward. Thus, our results suggest that prior mephedrone exposure differentially alters ethanol reward in adolescent and adult rats.

## 1. Introduction

Mephedrone (4-methylmethcathinone) is a synthetic cathinone derivative that is structurally and pharmacologically related to the psychostimulants (3,4-methylenedioxymethamphetamine (MDMA) and other amphetamines) [1] and has been classified as a new psychoactive substance (NPS) [2,3]. Mephedrone is abused by club drug users, predominantly adolescents and young adults [4,5], in a binge-like fashion (“stacking”) [6]. Published data indicate that most mephedrone users are engaged in “heavy” alcohol use immediately prior to consuming mephedrone, and then they reduce alcohol consumption as the effects of mephedrone are experienced during the drug episode [7]. Another study shows that mephedrone is commonly used simultaneously with alcohol (ethanol) by polydrug users [8]. Such drug combination produces more intense feelings of euphoria and well-being, compared with either drug alone [9]. Yet another study has revealed that past-year stimulant users (cocaine, amphetamine) appear to be more sensitive to ethanol stimulant properties than never-users. The effect was specific for stimulant drug users and never observed after cannabis or hallucinogen use [10]. It is, however, not clear whether more intense ethanol rewarding effects are observed in past-year mephedrone users.

The nucleus accumbens (NAc) is a major component of the ventral striatum (vSTR) and has been thought to be a key structure involved in mediating motivational and emotional processes, the limbic–motor interface, and the effects of certain psychoactive drugs. The NAc has been implicated in numerous neurological and psychiatric disorders, including drug abuse and addiction [11]. It receives extensive dopaminergic input from the ventral tegmental area (VTA). The NAc also receives glutamatergic input from the prefrontal cortex (PFC), amygdala, and hippocampus. Drugs of abuse stimulate dopamine (DA) release in the NAc, leading to dopamine D1 and D2 receptors (D1R and D2R) activation, which could regulate motivation for reward [12,13].

Mephedrone increases DA levels in the NAc, striatum, and frontal cortex in rats [14,15,16,17,18] and inhibits the reuptake of DA in the mesolimbic structures, including the NAc [15,16]. In addition, mephedrone clearly inhibits DA transporter (DAT) [16] and blocks in vitro DA re-uptake through the vesicular monoamine transporter-2 (VMAT2) in a dose-dependent manner [17]. Thus, an increase in extracellular DA levels following mephedrone exposure likely leads to aberrant DA signaling and stimulant-related behaviors. Ethanol is also able to increase burst firing of DA neurons in the NAc by direct (inhibiting gamma-aminobutyric (GABA)egric interneurons in the substantia nigra reticulata) (see [19] for a review) or indirect mechanisms [20,21,22].

Matrix metalloproteinases (MMPs), a family of proteolytic enzymes, can cleave extracellular matrix (ECM) proteins, which provide structural scaffolding to surrounding cells, to allow for the reconfiguration of neuronal pathways [23,24]. MMP-9 is a secreted zinc-dependent extracellular endopeptidase (gelatin protease) that can remodel the pericellular environment, primarily through the cleavage of ECM proteins [25] and cell-surface components. During development, MMP-9 can regulate local circuit reorganization through its ability to control synaptogenesis, axonal pathfinding, and myelination [26]. In the adult brain, MMP-9 is thought to play a role in adult neurogenesis and synaptic plasticity [27]. Dysregulation of MMP-9 activity has been reported after using drugs of abuse [28], including mephedrone [29,30]. Moreover, it has been reported that MMPs, mainly MMP-2 and MMP-9, are activated and play functional roles in such aspects of addiction, as motivation in mice and human subjects [31], rewarding effect [32,33,34], or relapse/reinstatement in rats [28,35,36].

By means of the conditioned place preference (CPP) paradigm, in the present study, we determined whether prior binge mephedrone administration during adolescence alters ethanol reward. Additionally, we examined whether age (adolescent vs. adult) and MMP-9 are important in this regard. In rodents, “adolescence” is considered as the period from postnatal day (PND) 25 to PND 60, which corresponds to early adulthood [37,38]. Adolescence is characterized by brain maturation, synaptic refinement, and myelination [39,40]. All these factors make the adolescent brain more sensitive to the deleterious effects of drug abuse and may lead to substance use disorders [37,39,40,41,42,43,44]. Furthermore, the current study sought to determine whether minocycline, an inhibitor of MMP-9 activity [45,46,47] can alleviate mephedrone-induced changes in the developing ethanol reward. Herein, in a set of biochemical experiments, in adolescent and adult rats, we assessed the level of MMP-9 and the expression of D1 receptor and Cav1.2 (a subtype of L-type calcium channels) after mephedrone and/or ethanol administration in the vSTR, a brain structure that is thought to be involved in reward processing [48]. In addition, we measured the expression of N-methyl-D-aspartate receptor (NMDAR) subunits (GluN1, Glu2A, Glu2B) in the vSTR of adult rats after the binge-like mephedrone administration in adolescence.

## 2. Results

### 2.1. Ethanol CPP

**Figure 1:**
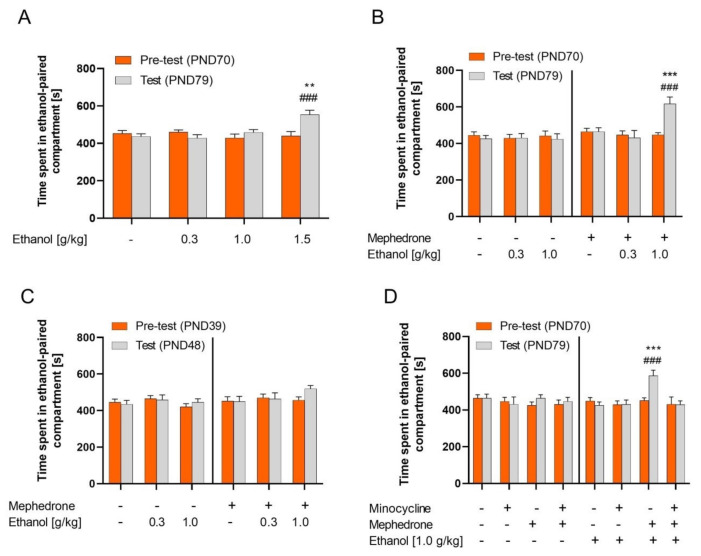
Effect of mephedrone and minocycline pretreatment on rewarding effect of ethanol measured in the CPP test. The data obtained in the CPP test are expressed as time in seconds (s) spent in the ethanol-paired compartment ± SEM in the pre-test and test. (**A**) Ethanol (1.5 g/kg, n = 7–8/group) alone induced CPP in adult (PND79) rats; ** *p* < 0.01 vs. EtOH 1.5 g/kg, pre-test; ^###^  *p* < 0.001 vs. NaCl 0.9%, test; (**B**) Ethanol (1.0 g/kg, n = 7–8/group) induced CPP in mephedrone-pretreated (3 × 10 mg/kg/day, PND30–36, n = 7–8/group) adult rats; *** *p* < 0.001 vs. mephedrone/EtOH 1.0 g/kg, pre-test; ^###^  *p* < 0.001 vs. 0.9% NaCl/EtOH 1.0 g/kg, test; (**C**) Ethanol (0.3, 1.0 g/kg, 15% *v*/*v*, i.p.) did not induce CPP in mephedrone-pretreated (3 × 10 mg/kg/day, PND30–36, n = 7–8) adolescent (PND48) rats; (**D**) Minocycline (45 mg/kg i.p.) pretreatment prevented mephedrone (3 × 10 mg/kg/day, PND30–36, n = 7–8/group) induced sensitivity to rewarding effect of ethanol (1.0 g/kg, 15% *v*/*v*, i.p.) given during conditioning in adult (PND79) rats; *** *p* < 0.001 vs. 0.9% NaCl/mephedrone/EtOH 1.0 g/kg, pre-test; ^###^ *p* < 0.001 vs. minocycline/mephedrone/EtOH 1.0 g/kg, test.

The two-way ANOVA of the CPP procedure (PND79) indicated the significant effect of ethanol dose (F (3, 56) = 4.215; *p* < 0.01) and an ethanol dose × time interaction (F (3, 56) = 6.348; *p* < 0.001). Post hoc analysis (Tukey’s test) showed that only animals treated with the dose of 1.5 g/kg of ethanol during conditioning induced significant CPP (Figure 1A). However, the post hoc test did not show any differences between groups on the pre-test day. Furthermore, one-way ANOVA did not indicate a significant effect of ethanol administration during conditioning on locomotor activity measured during the test (F (3, 28) = 0.8789; *p* > 0.05) (data not shown).

### 2.2. Effect of Mephedrone Pretreatment on Ethanol CPP in Adult and Adolescent Rats

Mephedrone pretreatment induced sensitivity to the rewarding effect of ethanol in adult rats (PND79). The three-way ANOVA revealed a significant effect of mephedrone pretreatment (F (1, 77) = 10.41; *p* < 0.01), ethanol dose (F (2, 77) = 3.847; *p* < 0.05), and interaction between mephedrone pretreatment × ethanol dose (F (2, 77) = 3.497; *p* < 0.05), ethanol dose × time (F (2, 77) = 3.688; *p* < 0.05), mephedrone pretreatment × time (F (1, 77) = 4.909; *p* < 0.05), and mephedrone pretreatment × ethanol dose × time (F (2, 77) = 4.681; *p* < 0.05). Post hoc analysis revealed that mephedrone pretreatment induced CPP in rats treated with ethanol at the dose of 1.0 g/kg (Figure 1B). In addition, two-way ANOVA did not indicate significant effects of mephedrone pretreatment during adolescence (F (1, 42) = 0.0060; *p* > 0.05) and ethanol administration during conditioning on locomotor activity measured during the test (F (2, 42) = 2.207; *p* > 0.05) (data not shown).

Three-way ANOVA revealed no significant effect of mephedrone pretreatment (F (1, 79) = 3.225; *p* > 0.05) on the rewarding effect of ethanol at the dose of 0.3 and 1.0 g/kg (F (2, 79) = 0.8173; *p* > 0.05) in adolescent rats (PND 48) (Figure 1C). A post hoc test did not show any differences between groups on the pre-test day. What is more, two-way ANOVA did not indicate significant effects of mephedrone pretreatment during adolescence (F (1, 42) = 0.005755; *p* > 0.05) and ethanol administration during conditioning on locomotor activity measured during the test (F (2, 42) = 0.3263; *p* > 0.05) (data not shown).

### 2.3. Influence of Minocycline Pretreatment on the Mephedrone Effect on the Ethanol CPP in Adult Rats

Minocycline, as an MMP-9 inhibitor, given before every mephedrone administration, attenuated the mephedrone induced sensitivity to ethanol rewarding effect in the CPP test in adult rats (PND79). Three-way ANOVA revealed the significant effect of minocycline/mephedrone pretreatment (F (3, 108) = 3.696; *p* < 0.05) and interaction between minocycline/mephedrone × ethanol (F (3, 108) = 3.813; *p* < 0.05) and minocycline/mephedrone × time (F (3, 108) = 3.941; *p* < 0.05). Post hoc analysis revealed that significant CPP was induced only in mephedrone but not minocycline/mephedrone-pretreated rats (Figure 1D). A post hoc test did not show any differences between groups in the pre-test day according to the unbiased CPP method.

Furthermore, two-way ANOVA did not indicate significant effects of minocycline/mephedrone pretreatment during adolescence (F (3, 56) = 0.1902; *p* > 0.05) and ethanol administration during conditioning on locomotor activity measured during the test (F (1, 56) = 0.07835; *p* > 0.05) (data not shown).

### 2.4. Biochemical Experiments

#### 2.4.1. Effect of Mephedrone Pretreatment, Ethanol Treatment, and Age of Rats on D1R Expression in the vSTR

**Figure 2:**
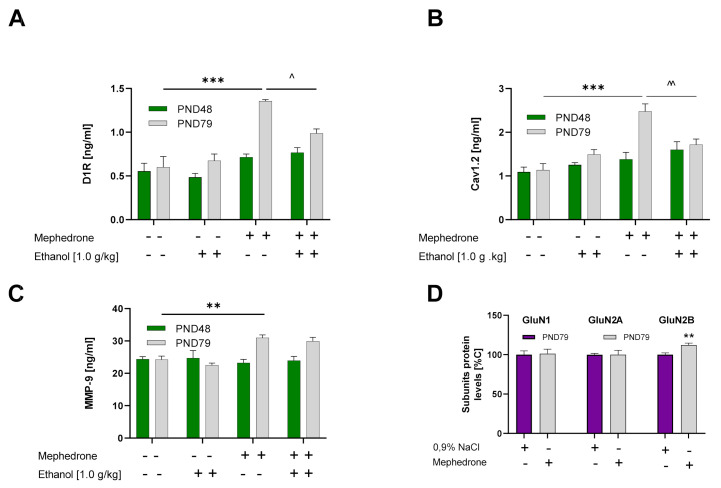
Effect of mephedrone (3 × 10 mg/kg/day, PND30-36, n = 6/group) pretreatment and ethanol (1.0 g/kg) treatment on: (**A**) D1R protein expression; *** *p* < 0.001 vs. 0.9% NaCl/0.9% NaCl%, PND79; ^ *p* < 0.05 vs. mephedrone/EtOH 1.0 g/kg, PND79. (**B**) Cav1.2 protein expression; *** *p* < 0.001 vs. 0.9% NaCl/0.9% NaCl%, PND79; ^^ *p* < 0.01 vs. mephedrone/EtOH 1.0 g/kg, PND79, (**C**) MMP-9 protein expression; ** *p* < 0.01 vs. 0.9% NaCl/0.9% NaCl%, PND79; in the vSTR of adolescent (PND48) and adult (PND79) rats. (**D**) Effect of mephedrone (3 × 10 mg/kg/day, PND30-36, n = 8/group) on expression of NMDA receptor subunits in vSTR of adult (PND79) rats; ** *p* < 0.01 vs. 0.9% NaCl. Corresponding membranes from Western blot analyses of NMDA receptor subunits and loading controls are included into Figure S2.

Three-way ANOVA showed a significant effect of mephedrone (F (1, 40) = 59.92; *p* < 0.001) and age of rats (F (1, 40) = 31.33; *p* < 0.001) but no ethanol effect (F (1, 40) = 5.545; *p* > 0.05). Moreover, statistical analysis revealed a significant effect of mephedrone × age interaction (F (1, 40) = 2.006; *p* < 0.01) and ethanol × mephedrone × age interaction (F (1, 40) = 8.439; *p* < 0.01) but no effect of other interactions on D1 receptor expression in the vSTR of rats that underwent the CPP procedure (Figure 2A).

Post hoc analysis revealed that mephedrone (*p* < 0.001) but not ethanol (*p* > 0.05) increased the D1 receptor expression level in the vSTR of PND 79 rats. Moreover, ethanol but not saline administration during conditioning decreased D1 receptor upregulation in adult rats (PND 79) pretreated with mephedrone during adolescence (*p* < 0.05).

#### 2.4.2. Effect of Mephedrone Pretreatment, Ethanol Treatment, and Age of Rats on Cav1.2 Expression in the vSTR

Three-way ANOVA showed a significant effect of mephedrone (F (1, 40) = 31.68; *p* < 0.001) but not ethanol (F (1, 40) = 0.0037; *p* > 0.05) and age of rats (F (1, 40) = 14.59; *p* < 0.001). Moreover, statistical analysis revealed the significant effect of a mephedrone × ethanol interaction (F (1, 40) = 7.384; *p* < 0.01), mephedrone × age (F (1, 40) = 5.750; *p* < 0.05), and mephedrone × ethanol × age of rat interaction (F (1, 40) = 9.041; *p* < 0.01) on Cav1.2 expression in the vSTR of rats that underwent the CPP procedure (Figure 2B).

Post hoc analysis revealed that mephedrone (*p* < 0.001) but not ethanol (*p* > 0.05) increased the Cav1.2 expression level in the vSTR. Moreover, ethanol administration decreased Cav1.2 expression, compared with animals that also previously received mephedrone, but not in animals that received saline during the CPP (*p* < 0.01) of PND79 rats.

#### 2.4.3. Effect of Mephedrone Pretreatment, Ethanol Treatment, and Age of Rats on MMP-9 Expression in the vSTR

Three-way ANOVA revealed a significant effect of mephedrone (F (1, 40) = 12.00; *p* < 0.01) and age of rats (F (1, 40) = 10.51; *p* < 0.01) and a mephedrone × age of rats interaction (F (1, 40) = 20.92; *p* < 0.001) but no effect of ethanol treatment (F (1, 40) = 0.2302; *p* > 0.05) on MMP-9 expression in vSTR of rats that underwent the CPP procedure (Figure 2C).

Post hoc analysis (Tukey’s multiple comparisons test) revealed that mephedrone (*p* < 0.01) but not ethanol (*p* > 0.05) increased the MMP-9 expression level in the vSTR of PND 79 rats. There were no significant changes in adolescent (PND48) animals.

#### 2.4.4. Influence of Minocycline Pretreatment on the Mephedrone Effect on MMP-9 Expression in the vSTR

Two-way ANOVA revealed a significant effect of minocycline (F (1, 24) = 11.23; *p* < 0.01) and mephedrone (F (1, 24) = 4.479; *p* < 0.05) but not a minocycline × mephedrone interaction (F (1, 24) = 3.521; *p* > 0.05).

Post hoc analysis (Tukey’s multiple comparisons test) revealed that mephedrone given during adolescence increased the MMP-9 expression level in the vSTR (*p* < 0.05) of adult rats. Minocycline given before mephedrone prevented this effect (*p* < 0.01) (Figure S1).

#### 2.4.5. Effect of Mephedrone Treatment on the Expression of NMDAR Subunits (GluN1, GluN2A, and GluN2B) in the vSTR

Statistical analysis revealed that mephedrone administration during adolescence induced significant changes only in the GluN2B subunit of NMDAR in the vSTR of adult rats (*p* < 0.01 by Student’s *t*-test; *p* = 0.0031) (Figure 2D).

## 3. Discussion

The current study provides evidence, for the first time, that adult rats with prior mephedrone experience express a more robust ethanol-induced CPP than their adolescent counterparts. The mephedrone effect in adult rats was not observed when minocycline, as an MMP-9 antagonist, was given before mephedrone administration. Along with these behavioral changes, we observed a substantial upregulation of D1R, Cav1.2, and MMP-9 expression in the vSTR in adult, but not adolescent rats with prior mephedrone administration. Moreover, upregulation of the GluN2B subunit of NMDAR was noted in adult rats with prior mephedrone administration. Ethanol administration during the conditioning sessions did not affect the MMP-9 level but reduced the upregulation of D1R and Cav1.2 in adult rats that had received mephedrone in adolescence. Together, these results suggest that prior mephedrone treatment differentially affected the rewarding action of ethanol in adolescent and adult rats.

Prior works have found differences in behavioral and neurochemical changes induced by amphetamine-type stimulants between adolescent and adult rodents [49,50,51,52]. Consistent with these studies, we found behavioral changes between adolescent and adult rats following ethanol conditioning with prior (binge) mephedrone administration during adolescence. Treated adolescent rats did not exhibit CPP preference following ethanol conditioning at the dose that induced robust CPP in adult rats (Figure 1). Moreover, no differences were observed in the locomotor activity of animals. Herein, the CPP response in adolescent but not in adult rats contrasts with a previous study showing that past stimulant users appear to be more sensitive to ethanol rewarding properties [10]. Unfortunately, we did not perform a dose–effect testing for ethanol CPP in adolescent rats. However, we assumed that adolescents show a shifted dose–effect curve. Thus, our experiment suggests that adolescent but not adult rats previously treated with mephedrone might need higher doses of ethanol (will drink more) to achieve the same response. Extending these results to humans, it is conceivable that adolescent mephedrone users may be at a greater risk of developing ethanol addictive behaviors than adults, similar to MDMA adolescent users [53]. Furthermore, due to enhanced reward experience, adults most likely chase the drug and develop addictive behaviors faster than adolescents.

The CPP response under a drug-free state (CPP expression) represents the motivational effects of the contextual cues that gain saliency following pairing with addictive drugs [54,55]. It appears that prior mephedrone exposure has no impact on the motivational valence of the context (associated with ethanol pairing) in adolescents but that it increases it in adult rats, as we observed a robust CPP in adult rats with prior mephedrone experience but not in adolescent rats. The CPP expression, on the other hand, is a representative of the memory retrieval of the conditioned response that is acquired due to pairing of the subjective effects of the drug and the context during conditioning [54,55]. This response is more robust in adult compared with adolescent rats with prior mephedrone administration. Although we expected to observe a greater response in adolescent rats, it appears that prior mephedrone exposure may differentially bring about molecular changes, thereby leading to these behavioral changes. These results suggest that mephedrone exposure during adolescence, similar to amphetamines exposure [56], may promote long-lasting neurophysiological alterations (remodeling) that influence their future behaviors, including increased reward sensitivity in adult but not adolescent rats.

Release of DA in the NAc is a key process related with the reinforcing and rewarding properties of a drug [57]. Because the NAc D1R is mainly involved in the acquisition of ethanol induced CPP [58], we evaluated the expression of this receptor in the vSTR in the adolescent and adult ethanol conditioned rats with/without prior mephedrone administration. We found that ethanol alone did not have significant impact on the expression of D1R and L-type Cav1.2 channels expression in the vSTR of adolescent or adult rats in the drug-free state during the CPP test (24 h after the last ethanol conditioning session). However, there was a significant upregulation of D1R in adult rats with prior mephedrone treatment. This mephedrone effect was accompanied by an upregulation of L-type Cav1.2 channels expression. Such effects were not observed in adolescent animals—(probably) because mephedrone-induced neurophysiological alterations were not sufficiently developed. Furthermore, ethanol administration during conditioning in adults increases burst firing of DA neurons in the NAc, see [19]; therefore, ethanol conditioning reduced the upregulation of D1R and Cav1.2 in the mephedrone-treated animals. These findings suggest that the dopamine system in adults with prior mephedrone administration was more vulnerable than adolescents to the rewarding effect of ethanol. Thus, even a minor ethanol-induced change/increase in extracellular dopamine was able to reduce the upregulation of D1R and Cav1.2 expression in the animals with prior mephedrone administration. The mechanism underlying the resistance to the effects of ethanol in adolescent rodents is not well understood. However, published data show that compared with adult rodents, adolescent rodents show reduced dopamine level in the NAc and PFC [52]. These findings provide evidence that dopamine responses to ethanol exposure during adolescence are/can be less intense than that induced by ethanol exposure in adults.

Published data suggest that MMP-2 and MMP-9 are involved in the regulation of methamphetamine (METH)-induced changes in DA release and uptake in the NAc [34,59]. Additionally, METH-induced behavioral sensitization and reward were markedly attenuated in MMP-2- and MMP-9-deficient mice, compared with those in wild-type [32]. The current study confirms the role of MMP-9 in the ethanol-induced reward in adult rats with prior mephedrone administration. Minocycline is a commonly used semi-synthetic tetracycline with anti-inflammatory and anti-apoptotic properties [60,61]. Minocycline interferes with MMP activity [62,63] and has been shown to be neuroprotective in cerebral ischemia [64] and in other models of brain injury [65,66]. Published data showed that minocycline inhibits enzymatic activity of gelatin protease, especially of MMP-9 [45,46,47]. Our results support its inhibitory effect on MMP-9 expression in the vSTR (Figure S1). Herein, minocycline given before mephedrone administration in adolescence reduced the rewarding effect of ethanol in adult rats. Furthermore, our findings indicate that binge mephedrone administration during adolescence induced upregulation of MMP-9 in the vSTR in adult but not in adolescent rats, which was abolished by minocycline pretreatment (see Figure S1). Considering these data, we hypothesized that mephedrone treatment during adolescence differentially affected the expression of MMP-9 in adolescent vs. adult rats.

Published data confirm that treatment with drugs of abuse increases MMPs activity in various brain structures [28,32,36,67]. However, data concerning ethanol are controversial. Some of the research has demonstrated that both acute and chronic intermittent ethanol exposure increased [68], but others [69] found decreased MMP-9 expression in various brain structures after ethanol injections. However, MMP-9 activity is increased in the brain of alcoholics, suggesting that the timing of measuring MMP activity after ethanol exposure may yield different results [70].

On the other hand, the acquisition of CPP for abuse drugs is a learning process [71]. Still, while MMP-9 is increased during drug-induced learning processes, when learning/plasticity is posited to have been completed, MMP-9 expression returns to pharmacological control levels in some brain structures, including the NAc [35,69,72,73,74]. Thus, we hypothesize that transient changes in MMP-9 could have occurred in the vSTR during the active phase (rising phase of the acquisition curve when performance is improving) of learning and memory consolidation of ethanol-induced CPP in rats. However, there were no changes in the MMP-9 level on the test day when this task had been established. These results suggest that on the test day the synaptic changes had been completed and that MMP-9 levels had returned to normal.

In contrast with ethanol effects, our findings indicate that binge mephedrone administration during adolescence induced marked changes in MMP-9 level in adult (PND79), but not adolescent (PND48) rats. These results confirm our previous findings [30], when significant upregulation of MMP-9 expression was noticed after a delay of 14 days and lasted up to 38 days following the last mephedrone administration, suggesting that MMP-9 might contribute to later occurring processes. Other researchers found that dopaminergic and glutamate transmission can affect the ECM that surrounds and stabilizes synapses [75,76,77,78]. Interestingly, prime candidates for ECM remodeling are extracellular proteases, including MMP-9 [79]. Published data reported that neuromodulation via D1-type dopamine receptor can induce ECM proteolysis specifically via NMDAR/NR2B activation and intracellular calcium signaling [80,81], hence taking part in synaptic plasticity (in striatal medial spiny neurons and frontal cortex neurons). Thus, we hypothesize that such a mechanism could be involved in our study in adult rats with prior mephedrone administration. However, future studies will be needed to explain the influence of ethanol CPP on the NMDAR subunits expression in prior mephedrone-exposed rats.

In summary, our results suggest that binge-like mephedrone administration during adolescence sensitized adult but not adolescent rats to the rewarding effects of ethanol. In adult rats, these changes are associated with the upregulation of D1R and NMDAR/Glu2B, as well as upregulation of L-type of calcium channel and MMP-9 expression in the vSTR. It seems that MMP-9 activation was responsible for these neuroplastic changes in reward pathways because the blockade of its activity by minocycline inhibits vulnerability to ethanol reward. The decrease in the ethanol rewarding action in adolescents, compared with adult rats, may suggest that this may be one cause of the dose escalation in chasing pleasure in this population. In turn, adults with prior mephedrone exposure more rapidly develop addictive behavior than adolescents.

## 4. Materials and Methods

### 4.1. Animals

Male Wistar rats (OMD, Lublin, Poland) weighing 100–135 g at the initial phase of study were used in our experiments, as this experiment is a follow-up to a previous study [30] in which only males were included. The results obtained from female rats may [82] or may not be [83] different because of estrous cycle stage. The experiments began at PND 30. The 234 animals (n = 7–8/group) were kept double-housed in cages (55 cm × 33 cm × 20 cm) under standard laboratory conditions such as a constant temperature of 22 ± 1 °C, controlled humidity within 55 ± 10%, natural day–night cycle (12/12 h), and free access to drinking water and standard laboratory chow (Sniff Spezialdiäten GmbH, Soest, Germany). Before the behavioral experiments, rats were handled for 5 min per day for 5 consecutive days. Experiments were conducted between 8:00 a.m. and 7:00 p.m. All procedures were approved by the Local Ethic Committee (No. 67/2019) and were carried out according to the National Institute of Health Guidelines for the Care and Use of Laboratory Animals and the European Community Council Directive of November 2010.

### 4.2. Drugs

Mephedrone hydrochloride (Tocris Bioscience, Bristol, UK) and minocycline hydrochloride (Pol-Aura, Dywity, Poland) were dissolved in sterile 0.9% saline (0.9% NaCl, Baxter, Warsaw, Poland). The solutions for administration were prepared ex tempore before each administration. Rats were treated intraperitoneally (i.p.) with mephedrone (10 mg/kg), 3 times a day according to the method described earlier [84] with minor modification. Minocycline (45 mg/kg) with confirmed neuroprotective properties [85], good tissue penetration, and long elimination half-life [86] was administered i.p. once daily, immediately before morning injection of mephedrone. Substances were administered for 7 consecutive days, beginning from PND 30.

### 4.3. CPP Apparatus

In the experiment, six identical rectangular boxes (54 cm × 35 cm × 49 cm) were used. Each was composed of two compartments with the same dimensions but differing in color of walls and texture of the floor. Between the compartments, there was a square passage closed with a guillotine door allowing, when opened, free movement between apparatus compartments. The walls of one were uniformly black, while the other was black and white vertically striped. The experimental room’s luminance was adjusted so that the environmental (visual and tactile) cues did not produce a significant baseline preference for one of both compartments. Between each paradigm procedure, the whole apparatus was cleaned with 10% ethanol solution to neutralize the odor cues. The apparatus was kept in a soundproof room with neutral noise masking and dim illumination (40 lx). The CPP performance was measured by computerized video tracking (VideoMot, TSE-Systems, Bad Homburg, Germany). Time spent in the compartment associated with ethanol was measured pre-test and test as measure of the degree of conditioning induced by the drug.

### 4.4. CPP Procedure

The CPP unbiased paradigm was performed according to the method described earlier [87] with minor modifications. It consisted of four phases that lasted for 11 consecutive days: habituation (1 day), pre-test (1 day), conditioning (8 days), test (1 day). Time spent in each compartment was measured during the pre-test phase to separate rats into groups with approximately equal biases for each compartment. An appropriate control group (0.9% NaCl-treated during all phases of experiments) was employed. This underwent the same CPP procedure as the drug-treated rats.

*Habituation*: On the first day, rats were placed in the random compartment for 15 min with free access to both rooms and could freely explore the apparatus.

*Pre-test*: On the second day, the procedure was the same, but computerized video tracking measured the time the rats spent in each apparatus compartment. The analysis showed no preferences. No drugs were administered during the first and the second day of this phase.

*Conditioning*: The animals were conditioned twice daily (morning and afternoon sessions) for eight consecutive days with at least a 3 h rest period between sessions. In the morning, the animals received ethanol (0.3, 1.0, or 1.5 g/kg, 15% *v*/*v*, i.p.), or equivalent volume of 0.9% NaCl, and were injected immediately prior to the conditioning session and then placed for 30 min into the drug-paired compartments with the guillotine doors closed. In the afternoon, all animals were injected with saline and placed into the drug-free compartment for 30 min.

*Test (CPP expression)*: The rats were confined individually in the apparatus and left for 15 min, having free access to both compartments. The animals were not injected during this phase. The amount of time spent by the animals in each compartment was measured and recorded. The locomotor activity of individual rats was measured as the distance traveled within 15 min of the test phase (CPP test).

#### 4.4.1. The Effect of Mephedrone Pretreatment on the Ethanol CPP

At PND30, the rats were injected with saline or mephedrone (10 mg/kg), three times a day for 7 consecutive days. Next, the animals were assigned into two cohorts. The first cohort was subjected to CPP procedure at PND38 (habituation (1 day, PND38), pre-test (1 day, PND39), conditioning (8 days, PND40-47), and test (1 day, PND48)).

The second cohort was subjected to the CPP procedure at PND69 (habituation (1 day, PND69), pre-test (1 day, PND70), conditioning (8 days, PND71-78), and test (1 day, PND79)). The following groups (n = 7–8/group) of animals were used in each cohort: 0.9% NaCl/0.9% NaCl; 0.9% NaCl/ethanol (0.3 g/kg); 0.9% NaCl/ethanol (1.0 g/kg); mephedrone/0.9% NaCl; mephedrone/ethanol (0.3 g/kg); mephedrone/ethanol (1.0 g/kg).

After the completion of the ethanol CPP test, the animals were decapitated, and the brain structures were removed and frozen (at −80 °C) for further neurochemical analyzes.

#### 4.4.2. The Influence of Minocycline Pretreatment on the Mephedrone Effect on the Ethanol CPP

This part of the study was performed to indicate whether MMP-9 is involved in mephedrone-induced effects. For this purpose, minocycline, an inhibitor of MMP-9 [48], was given at the dose of 45 mg/kg [75] during mephedrone administration (n = 7–8/group), every day before first drug injection (PND30-36). Next, the animals (PND69) were subjected to the CPP procedure according to the method described above, with the exception that only one dose of ethanol (1.0 g/kg) was given during conditioning. After CPP procedure, one group of animals was decapitated, and the dissected brain structures were subjected to biochemical experiments to evaluate the influence of minocycline and mephedrone on MMP-9 expression in the vSTR.

#### 4.4.3. The Ethanol CPP

A separate group of rats was subjected to the CPP procedure (PND69) with saline pretreatment (PND30-36). Ethanol was given at the dose of 0.3, 1.0, or 1.5 g/kg, 15% *v*/*v*, i.p. during conditioning to indicate whether ethanol alone induced the rewarding effect in CPP.

### 4.5. Biochemical Experiments

#### 4.5.1. ELISA Assay

Quantitative measurement of MMP-9, D1R, and Cav1.2 in such rat brain structures as the vSTR was performed using a Rat MMP-9 ELISA Kit (Reddot Biotech, Kelowna, BC, Canada), a Rat Dopamine Receptor D1 (DRD1) ELISA kit (Reddot Biotech, Kelowna, BC, Canada), and Rat Voltage-dependent L-type Calcium Channel Subunit Alpha-1C ELISA Kit (Bioassay Technology Laboratory, Shanghai, China), respectively, following manufacturers’ protocols. Firstly, frozen brain structures were homogenized in cold buffer at pH 7.4 (0.32 M sucrose, 1 mM HEPES, 1 mM MgCl_2_, 1 mM NaHCO_3_, and 0.1 mM PMSF) containing cocktails of protease and phosphatase inhibitors (Sigma-Aldrich, Saint Louis, MO, USA), using a homogenizer ball (Bioprep-24, Allsheng, China) (10 s at 10,000 rpm). Then, homogenates were centrifuged for 5 min at 5000× *g*, the supernates were immediately removed, and protein concentration in the supernates was measured using a bicinchoninic acid assay (BCA) protein assay kit (Serva, Heidelberg, Germany). From each sample, 100 µg of protein was used in each ELISA assay. All data are expressed in ng/mL.

#### 4.5.2. Western Blot

An additional group of adult rats (n = 48) that received mephedrone during adolescence was decapitated on PND79. The vSTR was dissected and subjected to NMDAR subunit assessment by the Western blot test. The frozen brain structure was homogenized under the same conditions as in Section 4.5.1. Protein concentration in the homogenates was measured using a bicinchoninic acid assay (BCA) protein assay kit (Serva, Heidelberg, Germany). Homogenate (10 μg of protein) was then denatured, resolved by 10% SDS polyacrylamide gels, and transferred to a polyvinylidene difluoride (PVDF) membrane. Next, membranes were blocked in 3% non-fat dry milk, and separate sets of membranes were probed with mouse anti-GluN1 monoclonal antibody (1:1000; 32-0500, Thermo Fisher Scientific, Waltham, MA, USA), rabbit anti-GluN2A polyclonal antibody (A-6473; 1:1000; Thermo Fisher Scientific, Waltham, MA, USA), and rabbit anti-GluN2B polyclonal antibody (1:1000; ab65783; Abcam, Cambridge, UK). β-actin was used as a control protein (mouse monoclonal antibody; 1:1000; A5441; Sigma-Aldrich, Saint Louis, MO, USA). Blots were washed and incubated with donkey goat anti-rabbit secondary antibody (1:6000; 926-68071; Li-Cor, Lincoln, NE, USA) or goat anti-mouse (1:6000; 926-32210; Li-Cor, Lincoln, NE, USA) and visualized using fluorescence detection Odyssey Clx (Li-Cor, Lincoln, NE, USA). Analysis was performed by Image Studio v.2.1. All data are expressed as percentage (%) of control.

### 4.6. Statistical Analysis

The data obtained in the CPP procedure are expressed as mean ± SEM of time spent in drug-paired compartment. The CPP results were evaluated by a three-way analysis of variance (ANOVA), followed by Tukey’s multiple comparisons test in order to compare differences between groups. Two-way analysis of variance (ANOVA) with the Tukey’s post hoc test was used to analyze the effect of mephedrone pretreatment, ethanol treatment, and age of rats on MMP-9, D1R, and Cav1.2 expression in the rats’ vSTR and the influence of minocycline on MMP-9 expression in mephedrone exposed rats. Unpaired Student’s *t*-test was applied to evaluate the effect of mephedrone treatment on the expression of NMDAR subunits in the rats vSTR.

## Data Availability

The data presented in this study are available on request from the corresponding author.

## Supplementary Materials

The following supporting information can be downloaded at: https://www.mdpi.com/article/10.3390/ijms23042122/s1.
