# Supplementary material for: Alteration of Ethanol Reward by Prior Mephedrone Exposure: The Role of Age and Matrix Metalloproteinase-9 (MMP-9)
Source: Int J Mol Sci. 2022 Feb 14;23(4):2122. doi: 10.3390/ijms23042122 (PMC8877998; doi:10.3390/ijms23042122)
Supplement: Supplementary file 1 [file ijms-23-02122-s001.zip › ijms- 1583919supplementary for conversion.pdf]

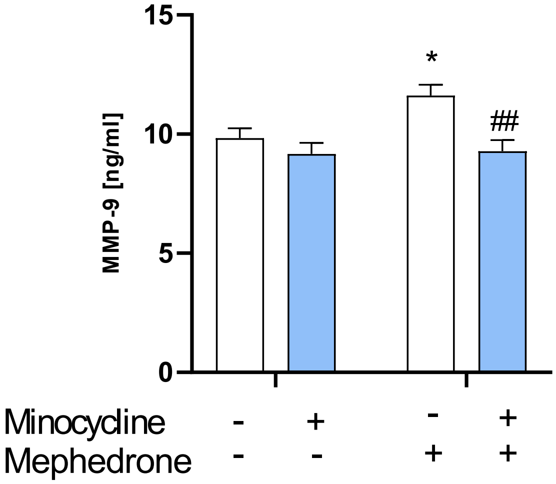

Figure S1. Influence of minocycline (45 mg/kg i.p.) pretreatment on the mephedrone (3 x 10 mg/kg/day x 7 days; PND30-36, n = 7/group) effect on MMP-9 expression in the vSTR of adult rats (PND79); *p < 0.05 vs. 0.9 % NaCl; ##p < 0.01 vs. mephedrone.

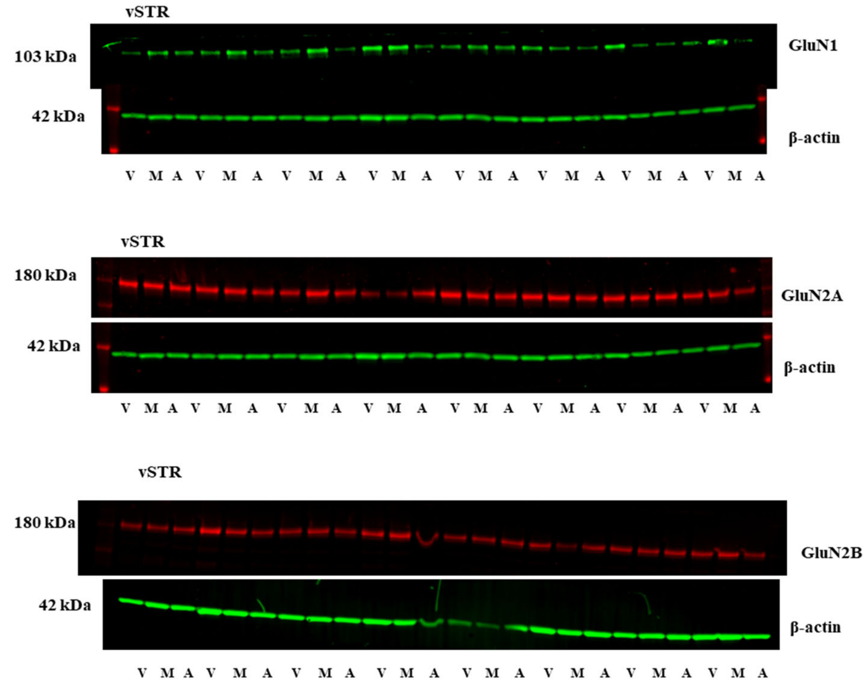

Figure S2. Corresponding membranes from Western blot analyses of NMDA receptor subunits and loading controls in the vSTR. V- vehicle, M- mephedrone, A- amphetamine (data not included in this paper). Control protein (β-actin) for GluN1 and GluN2A was the same.
